# Alendronate partially rescues the periodontal defects in OIM mouse model of osteogenesis imperfecta

**DOI:** 10.1038/s41598-024-84756-8

**Published:** 2025-01-02

**Authors:** Po-Jung Chen, Ke Wang, Meng-Hsuan Lin, Radhika Sharma, Zana Kalajzic, Mara O’Brien, Sumit Yadav

**Affiliations:** 1 Department of Growth and Development, University of Nebraska Medical Center, 4000 East Campus Loop South, 68583-0740 Lincoln, NE US; 2 Division of Orthodontics, University of Connecticut Health Center, Farmington, US; 3 Department of Adult Restorative Dentistry, University of Nebraska Medical Center, Lincoln, US; 4 University of Connecticut, Storrs, US; 5 University of Connecticut Health Center, Farmington, US

**Keywords:** Alendronate, Osteogenesis Imperfecta, Periodontal ligament, Sharpey’s fiber, Orthodontic tooth movement, Developmental biology, Health care

## Abstract

Osteogenesis imperfecta (OI) is a fairly common generalized connective disorder characterized by low bone mass, bone deformities and impaired bone quality that predisposes affected individuals to musculoskeletal fragility. Periodontal ligament (PDL)-alveolar bone and PDL-cementum entheses’ roles under OI conditions during physiological loading and orthodontic forces remain largely unknown. In addition, bisphosphonates (e.g., alendronate) are commonly used therapeutics for the treatment of OI. Our knowledge, in terms of the affects of alendronate treatment on the PDL entheses in OI is also far from complete. In this study, we identified craniofacial skeletal defects in an osteogenesis imperfecta (*oim*) murine model of OI. Relative to wild-type littermates, *oim* mice were found to have decreased skull length, cranial height/width/length, nose length, nasal length, and frontal length. Next, we discovered that *oim* mice exhibited defects in several dental structures, including short roots and decreased volumes of the alveolar bone, dentin, and cellular cementum. Further, we specifically investigated periodontal defects in the *oim* mice. Alveolar bone loss in *oim* mice was primarily associated with elevated bone resorption due to an increased osteoclast number, along with reduced bone formation related to increased sclerostin (SOST) expression. PDL fibers in *oim* mice were disrupted and discontinuous, while Sharpey’s fibers at the PDL-bone entheses were reduced. Mechanism-based studies showed that catabolism of the PDL was elevated in *oim* mice, as revealed by an increase in MMP13 and CTSK expression. Meanwhile, the quality of the collagen fibers were impaired in *oim* mice due to a large accumulation of uncleaved collagen I fibers. With alendronate treatment, however, we could partially rescue these phenotypes. This study, for the first time, characterized periodontal defects in *oim* mice, detailed craniofacial defects and demonstrated the effectiveness of alendronate in partially restoring these defects.

## Introduction

Osteogenesis Imperfecta (OI), also known as “brittle bone disease”, is a heterogeneous disease of the connective tissue characterized by a mild to severe defect in bone matrix that leads to bone deformity and repeated fractures^1–3^. OI has a cumulative incidence of approximately 1 in 15,000–20,000 individuals^4^. The majority of OI cases (85–90%) are caused by quantitative mutations, or mutations altering the structure of *COL1A1* and *COL1A2*, coding for the α1(I) and α2(I) chains of type I collagen, the most abundant protein of bone, skin and tendon extracellular matrices^1,4^.

Orthodontic treatments are often required in patients with OI due to a higher prevalence of craniofacial and dental abnormalities. According to the Dental Health Component-Index of Orthodontic Treatment, the need index was found to be 88.5% for the OI group, compared to 24.8% for the healthy control group; and the dental aesthetic index was found to be 61.5% and 51.8% in the OI and control group, respectively^5^. In addition, a Class III occlusal relationship was found in a high percentage (63%) of patients with OI, and the maxilla was more retrusive than the mandible in relation to the cranial base^6,7^. Furthermore, OI is associated with an increased prevalence of anterior and posterior crossbite, anterior and posterior open bite, hypodontia, and dentinogenesis imperfecta^5,8,9^. Orthodontic treatment, with or without orthognathic surgery, is often necessary to help patients with OI achieve proper occlusal function, stability, and aesthetics^8,10,11^. However, our knowledge remains far from complete in terms of understanding periodontium remodeling during orthodontic tooth movement (OTM) in OI.

OI is best managed by a multidisciplinary approach including physiotherapy, rehabilitation, pharmacological treatment, and orthopedic surgery^1,3,12^. Bisphosphonates, a type of antiresorptive medication, are widely used to treat OI^1,3,12^. The aim of bisphosphonate treatment is to increase bone volume in cases of classic OI (characterized by fragile bones which fracture easily), however the newly formed bone still contains defective collagen. The hypothesis behind this treatment strategy is that an increase in the volume of bone (even of impaired quality) would be beneficial to bone strength^1,12^. Alendronate, commonly used for treatment of OI, is in the family of second- and third-generation bisphosphonates which have nitrogen-containing R^2^ side chains. Alendronate binds to bone surfaces with high affinity, inducing osteoclast endocytosis from the bone surface leading to farnesyl pyrophosphate synthase (FPPS) inhibition and osteoclast apoptosis^13^. In fact, studies have demonstrated that bisphosphonates, such as alendronate, can improve bone microarchitecture, bone mass, long bone bowing deformity, and restore vertebral size and shape in OI patients^12,14–18^. However, compared to numerous studies on long bones, the effects of bisphosphonates on craniofacial bone and periodontium have been insufficiently studied. Furthermore, the mechanism by which bisphosphonates affect the periodontal tissue remodeling process under physiologic load in patients with OI is still unknown, thus making orthodontic treatment in OI patients unpredictable.

Therefore, in this study our aim was to characterize periodontal tissue using the *oim* murine model. In addition, we characterized craniofacial defects in these mice and determined the biological responses of the PDL entheses under physiologic loads, with- or without- alendronate treatment.

## Materials and methods

### Mice

*Oim* homozygous mice show a disease phenotype which is similar to the naturally occurring mutation causing human type III OI^19^. The mutation in *oim* mice is a single nucleotide deletion (G) which alters the terminal approximately 50 amino acids of the pro-alpha 2 C-propeptide and prevents association with the pro-alpha1 chains. Mice homozygous for *oim* exhibit osteopenia, progressive skeletal deformities, fractures, cortical thinning, and reduced body size^2,19,20^. In this study, we used male mice homozygous for the *oim* mutation (Jackson Laboratory, Bar Harbor, ME stock # 001815, C57BL/6J background), as well as male wild type (WT) littermates. The mice were housed in a temperature-controlled environment with 12-h light/dark cycles. Four groups (10 mice/group) were assigned: (1) WT mice control (WTC), (2) WT mice with alendronate administration (WTA), (3) *oim* mice control (OIC), (4) *oim* mice with alendronate administration (OIA).

The housing, care, and experimental protocols for this study were reviewed and approved by the Institutional Animal Care and Use Committee (IACUC) at the University of Connecticut School of Dental Medicine. All methods were performed in accordance with the relevant guidelines and regulations. This study is reported in accordance with ARRIVE guidelines.

## Alendronate and fluorochrome injection

- Alendronate sodium trihydrate (Sigma-Aldrich, St. Louis, MO) was diluted to a stock solution of 10 mg/mL in water, aliquoted, and stored at minus twenty degrees Celsius. From this stock solution, we prepared a working solution at a concentration of 25 ug/mL saline, which was used for treatment of mice. Mice were treated at either 4 weeks- or 3 months- of age, with alendronate by subcutaneous injection (0.05 mg/kg) as described previously^21^ on the initial day and every other day for 2 weeks, followed by euthanasia with carbon dioxide.
- To determine new bone formation, fluorochromes were administered intraperitoneally as described previously^22,23^. Calcein (Fluka, Arlington, VA) was prepared at a concentration of 3 mg/ml in 2% NaHCO3 pH 7.4, stored at minus twenty degrees celsius, and administered by intraperitoneal injection at a dose of 10 mg/kg body weight at 7 days prior to euthanasia. Alizarin complexone (Sigma-Aldrich, St. Louis, MO) was prepared at a concentration of 10 mg/ml in 2% NaHCO3 pH 7.4, stored at minus twenty degrees Celsius, and administered by intraperitoneal injection at a dose of 20 mg/kg body weight at 2 days prior to euthanasia.

## Sample Preparation

Skulls of experimental mice were collected and fixed in 4% paraformaldehyde in phosphate-buffered saline (PBS, pH = 7.4) at 4 °C for 2–4 days and used for micro-CT scans. Mandibles were then dissected and analyzed using micro-computerized tomography (µCT).

For each animal, mandibles (non-decalcified) from one side were dehydrated in ascending graded ethanol (EtOH) (75%, 95%, two rounds of 100%, 2–4 days each) followed by xylene, and embedded in methyl-methacrylate (MMA, Buehler, Lake Bluff, IL) as previously described^24^. The mandibles from the other side were decalcified in 15% EDTA at 4 °C, embedded in paraffin and cut into 4 μm-thick Sect^25^.

## Histomorphometry and immunohistochemistry

Decalcified paraffin sections were used for Sirius red, Masson’s trichrome, and TRAP staining as previously described^26,27^. The following antibodies were used for immunohistochemistry: goat polyclonal anti-sclerostin (1:100, R&D Systems, Minneapolis, MN, AF1598), goat polyclonal anti-periostin (1:100, R&D Systems, AF2955), goat polyclonal anti-sclerostin (SOST, 1:100, R&D Systems, AF1589), mouse monoclonal anti-cathepsin-K (CTSK, 1:100, Santa Cruz Biotechnology, Dallas TX, sc-48353), rabbit polyclonal anti-matrix metalloproteinase 13 (MMP13, 1:100, Abcam, ab39012), rabbit collagen I C-telopeptide (1:1000, Kerafast Inc., Boston, MA, LF-68), and rabbit collagen I C-propeptide (1:1000, Kerafast, LF-42). Corresponding Alexa Fluor 488 or 568 secondary antibodies (1:200, Thermo Fisher Scientific, Waltham, MA) were used for immunostaining^28^. Histology and immunofluorescence images were captured using a Zeiss Axioscan 7 scanning microscope^29^.

## Fluorochrome and FITC imaging

Thick Sects. (300–400 μm) were cut from the non-decalcified MMA-embedded blocks with a diamond bladed saw (Buehler, Lake Bluff, IL), ground down to a final thickness of 30–50 μm, and polished for confocal imaging^22^. Fluorescein isothiocyanate (FITC) is a low molecular weight dye that fills in spaces which have little or no mineral content, but does not enter the mineralized matrix. Thus, the dye provides a visual representation of porous structures in the bone, including the lacunocanalicular system^23,30^. To prepare the 1% FITC solution, FITC powder (Sigma-Aldrich, F7250) was diluted in 100% EtOH, gently mixed using a magnetic stirrer overnight at room temperature until clear, and then filtered (0.2 μm membrane). Following dehydration in ascending graded EtOH, the non-decalcified bone slices were then placed in the freshly prepared FITC solution for 1–3 days (depending on the sample size) and kept in the dark at room temperature. The samples were washed in 100% EtOH for 24 h, followed by the subsequent steps of the MMA-embedding process. These FITC-stained plastic blocks were then cut and ground down to thin sections of 30–50 μm for confocal imaging, as described above.

### µCT and Radiograph

Micro-computerized tomography (µCT) analyses of the skulls and mandibles were performed using a Scanco µCT37 (Scanco Medical AG, Bassersdorf, Switzerland)^26,31^. X-ray tube potential of 55 kV and intensity of 145 µA were applied when scanning the samples. The voxel size was 5 μm and integration time was 700 ms.

The STL files of skull µCT scans were imported into MeshLab software and assessed for craniofacial skeletal linear measurements^32^, including cranial height, cranial width, cranial length, inner canthal length, nose length, nasal length, frontal length, parietal length, and skull length.

The regions of interest (ROI) for quantification of the dental structures are as follows: (1) alveolar bone: all the alveolar bone under the bifurcation of the mandibular first molar (M1); (2) dentin: dentin of the mesial root of M1; (3) enamel: all the enamel of M1; (4) cellular cementum (CC): CC of the mesial root of M1; (5) PDL: the PDL space surrounding the mesial root of M1. After contouring the ROI on the micro-CT scans, the following variables were measured: alveolar bone total volume (mm^3^), alveolar bone volume (mm^3^), mineralized volume per total volume (bone volume fraction) BV/TV (%), hydroxyapatite (HA) -specific bone mineral density (BMD) (mg/ccm HA), enamel volume (mm^3^), dentin volume (mm^3^), dentin mineral density (mg/ccm HA), cellular cementum volume (mm^3^), cellular cementum density (mg/ccm HA), and the PDL volume (mm^3^).

Following micro-CT analysis, radiographic images of the mandibles were collected using a Faxitron MX-20 Cabinet X-ray System (Faxitron X-Ray LLC, Lincolnshire, IL), prior to mounting for histological analysis.

### Statistical analysis

Data were analyzed with SPSS software (version 16.0; SPSS, Chicago, IL). All results were expressed as the mean ± standard deviation. Independent t-tests were used to compare the linear measurements of the skulls of the OIC and WTC groups. Two-way ANOVA tests with Tukey’s post-hoc test, were used to compare dental structures, root length, and bone labeling among the groups (OIC, WTC, OIA, WTA). The level of significance was set at *P* < 0.05.

## Results

### *Oim*^−/−^ mice exhibit persistent craniofacial skeletal and dental defects

**Fig. 1:**
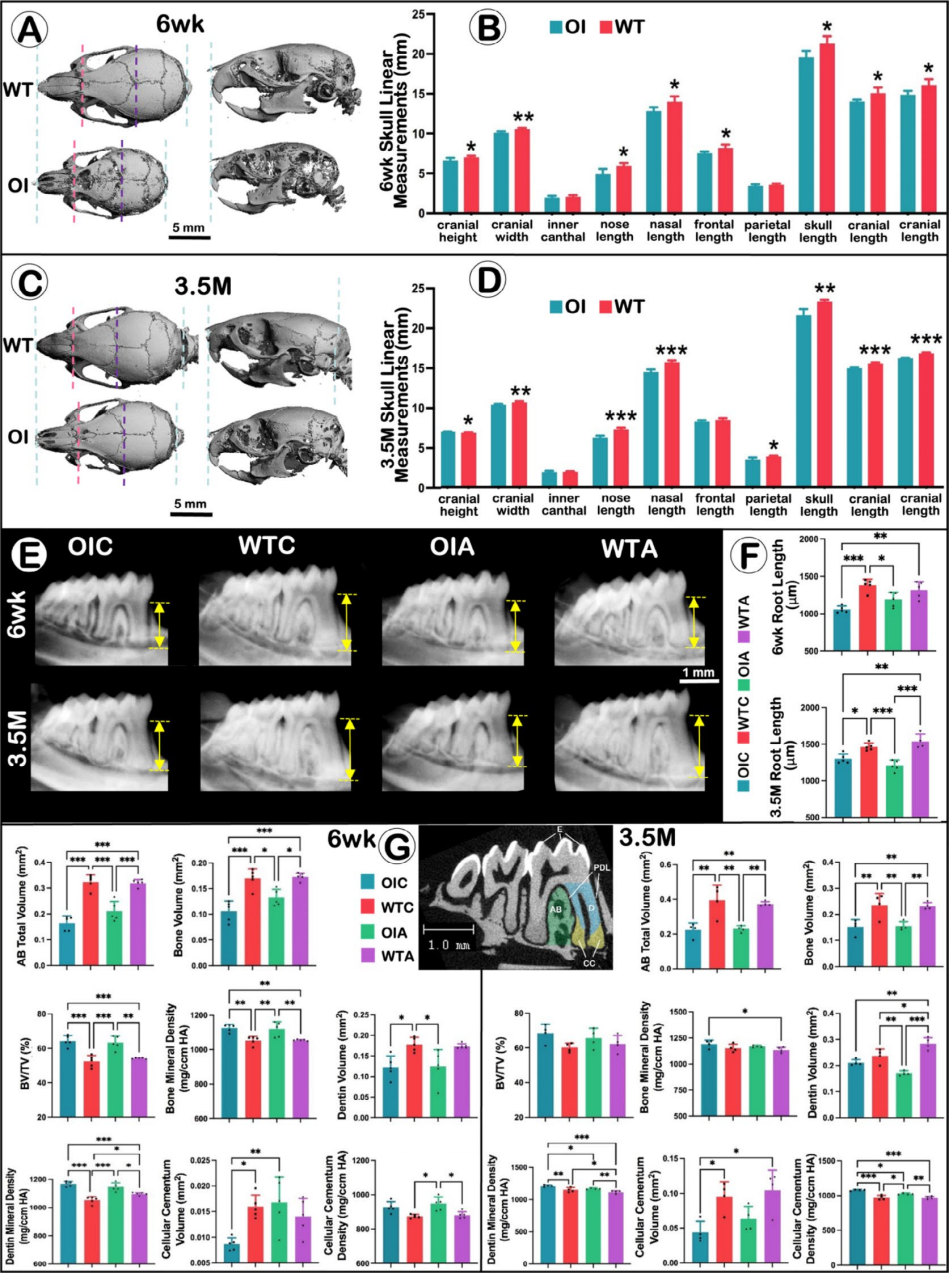
*Oim* ^*−/−*^ *Mice Exhibit Persistent Craniofacial Skeletal and Dental Defects*. (**A**) Representative 3D-µCT views of skulls from the 6-week old groups of *oim* and WT mice. (**B**) Quantification of linear measurements of skulls from the 6-week old groups using µCT. *n* = 5; Independent t-test between *oim* and WT mice, *P < 0.05, **P < 0.01. (**C**) Representative 3D-µCT views of skulls from the 3.5-month old *oim* and WT groups. (**D**) Quantification of linear measurements of skulls from the 3.5-month groups using µCT. *n* = 5; Independent t-test between *oim* and WT mice, *P < 0.05, **P < 0.01, ***P < 0.001. (**E**) Representative X-ray of mandibular teeth. Arrows indicate the length of the mesial root of the mandibular first molar. (**F**) Quantification of the mesial root length of the mandibular first molar. *n* = 4–5; Two-way ANOVA with Tukey’s post-hoc test, *P < 0.05, **P < 0.01, ***P < 0.001. (**G**) 2D sagittal sections from µCT scans showing the Region of Interest (ROI) for quantifying the enamel (E), alveolar bone (AB), dentin (D), and cellular cementum (CC) (middle panel). Quantification of dental structures from the 6-week old (left panel) and 3.5-month old (right panel) groups of *oim* and WT mice. *n* = 4–5; Two-way ANOVA with Tukey’s post-hoc test, *P < 0.05, **P < 0.01, ***P < 0.001.

*Oim* mice were found to have a smaller body mass than their WT littermates. Micro-CT analysis revealed that OI mice have a smaller skull size than WT mice in both the 6-week and 3.5-month-old groups. Further, 6-week-old OI mice also exhibited a Class III malocclusion with maxillary molars posterior to mandibular molars. Quantification of the µCT scans for *oim* mice showed a statistically significant reduction in several absolute inter-landmark distances, relative to WT animals, including reduced skull length, cranial height/width/length, nose length, nasal length, and frontal length (*P* < 0.05) (Fig. 1A-D).

Radiographic images were used to measure the root length of the M1 mesial root, and *oim* mice showed statistically significant reduction in root length, compared to WT mice. However, following alendronate administration, this phenotype was partially rescued (Fig. 1E, F).

µCT scans of the mandibles were used for measurement of dental structures. Quantitative analyses confirmed that *oim* mice have significantly decreased alveolar bone volume and cellular cementum in both the 6-week and 3.5-month-old groups. Dentin volume was also reduced in the group treated at 6-weeks of age. Alendronate administration partially rescue defects in the cellular cementum in *oim* mice (Fig. 1G).

### *Oim*^−/−^ mice display alveolar bone defects which can be partially improved by alendronate treatment

**Fig. 2:**
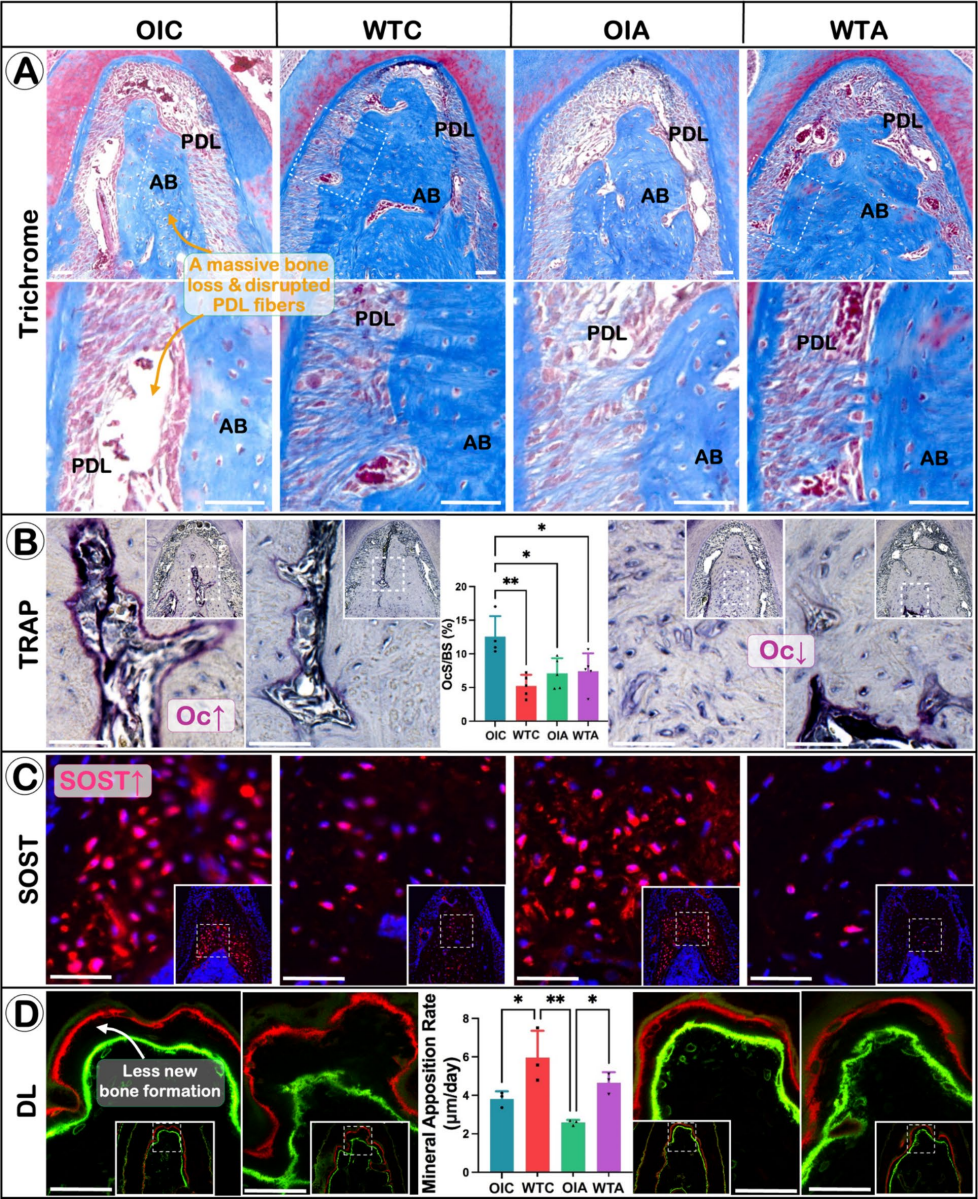
*Oim* ^−/−^ mice display alveolar bone defects which can be partially improved by alendronate treatment. (**A**) Representative Masson’s trichrome staining of mandibular first molars. (**B**) TRAP staining and quantification showing the percentage of osteoclast positive surface versus bone surface (OcS/BS). *n* = 4–5; Two-way ANOVA with Tukey’s post-hoc test, *P < 0.05, **P < 0.01, ***P < 0.001. (**C**) SOST immunostaining. (**D**) Representative images showing fluorescence labeling; Calcein (green label) and alizarin complexone (red label) were injected 7 days and 2 days before sacrifice, respectively. Mineral apposition rate was calculated by dividing the distance between two labels by 5 days (µm/day) (middle panel). *n* = 4–5; Two-way ANOVA with Tukey’s post-hoc test, *P < 0.05, **P < 0.01.

Consistent with the µCT analysis, trichrome staining also revealed severe alveolar bone loss in *oim* mice compared to WT, however, this bone loss could be partially rescued by alendronate administration. TRAP staining showed a dramatic increase in osteoclast activity in *oim* mice compared to WT, and this increased osteoclastic activity was almost completely inhibited by alendronate. Quantification of the ratio of osteoclast positive surface to bone surface (OcS/BS) further confirmed that alendronate restores osteoclastic activity (Fig. 2A, B; Fig. S2 A, B).

SOST, a secreted glycoprotein, is mainly produced by mature osteocytes, and inhibits bone formation through interactions with the Wnt/β-catenin signaling pathway^33–35^. We observed that the alveolar bone in *oim* mice exhibited increased levels of SOST expression, suggesting the catabolic effects of SOST played a critical role in bone loss in the *oim* mice. Interestingly, alendronate administration did not significantly affect SOST expression(Fig. 2C; Fig. S2 C).

To mark new bone formation, calcein and alizarin complexone were administrated to mice at 7 days, and 2 days before euthanasia, respectively. The distance between these two fluorescent dyes in the *oim* mice was much shorter than that in WT mice, indicating a decreased rate of new alveolar bone formation in *oim* relative to WT mice. Alendronate administration did not significantly affect the new bone formation rate (Fig. 2D).

### *Oim*^−/−^ mice displays a severe PDL phenotype which can be partially improved by alendronate treatment

**Fig. 3:**
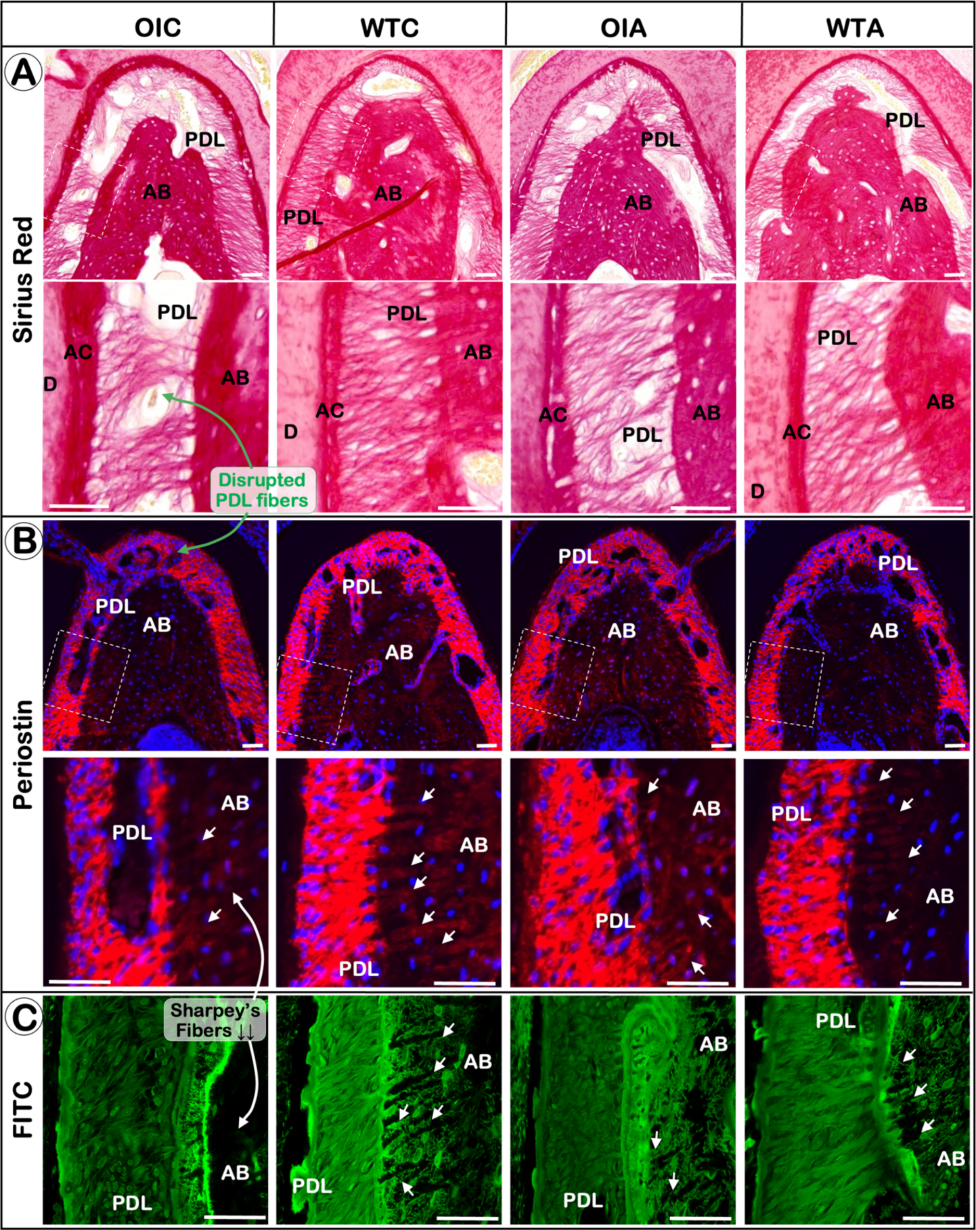
*Oim* ^−/−^ mice display a severe PDL phenotype which can be partially improved by alendronate treatment. Representative images of Sirius red staining (**A**), immunofluorescent staining of periostin (**B**) and FITC staining (**C**).

*Oim* mice showed increased discontinuity of the PDL fibers, and a great reduction of collagen fibers in the PDL and Sharpey’s fibers at the PDL-bone entheses when compared to WT mice, as shown by periostin immunostaining and Sirius Red staining. The disruption of the PDL and Sharpey’s fibers was found to be partially restored by alendronate treatment (Fig. 3; Fig. S3).

### Uncleaved procollagen I accumulation in the PDL of *oim*^−/−^ mice

**Fig. 4:**
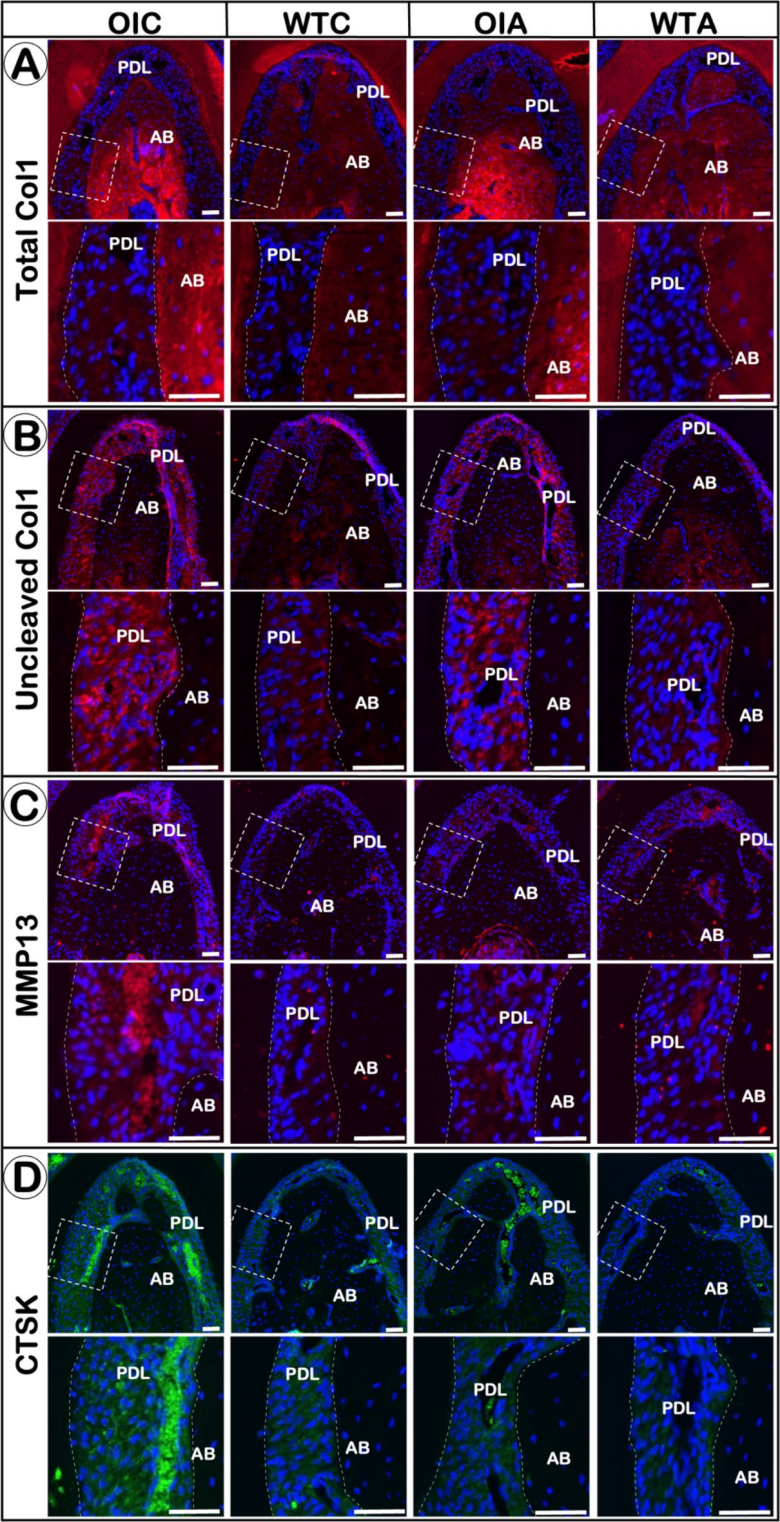
Molecular changes in the periodontium of *Oim* ^−/−^ mice. Representative images of immunofluorescent staining showing total Col1 (**A**), uncleaved Col1 (**B**), MMP13 (**C**) and CTSK (**D**) expression in PDL and alveolar bone tissue.

In order to investigate the mechanisms underlying malformed collagen fibers, observed in the *oim* mice, and the effects of alendronate, we examined collagen degradation and collagen quality. First, we identified significantly increased MMP13 and CTSK expression in the PDL of *oim* mice, indicating increased collagen degradation in the PDL of *oim* mice compared to WT. Next, we demonstrated that *oim* mice displayed a higher level of Col I C-propeptide in the PDL and alveolar bone, indicating a large accumulation of uncleaved procollagen I and decreased cleavage enzyme activity (i.e. the antibody against α1(I) chain C-propeptide only recognized uncleaved immature forms of Col I) in the *oim* animals. Meanwhile, the total collagen 1 was also higher in *oim* mice than in WT (i.e. the antibody against α1(I) chain C-telopeptide recognized all forms of Col I [Total Col I])(Fig. 4; Fig. S4).

With alendronate treatment, MMP13 and CTSK levels in the PDL were reduced, although total Col1 and uncleaved Col1 seemed unchanged.

### *Oim*^−/−^ Mice also exhibit defects in cementogenesis

**Fig. 5:**
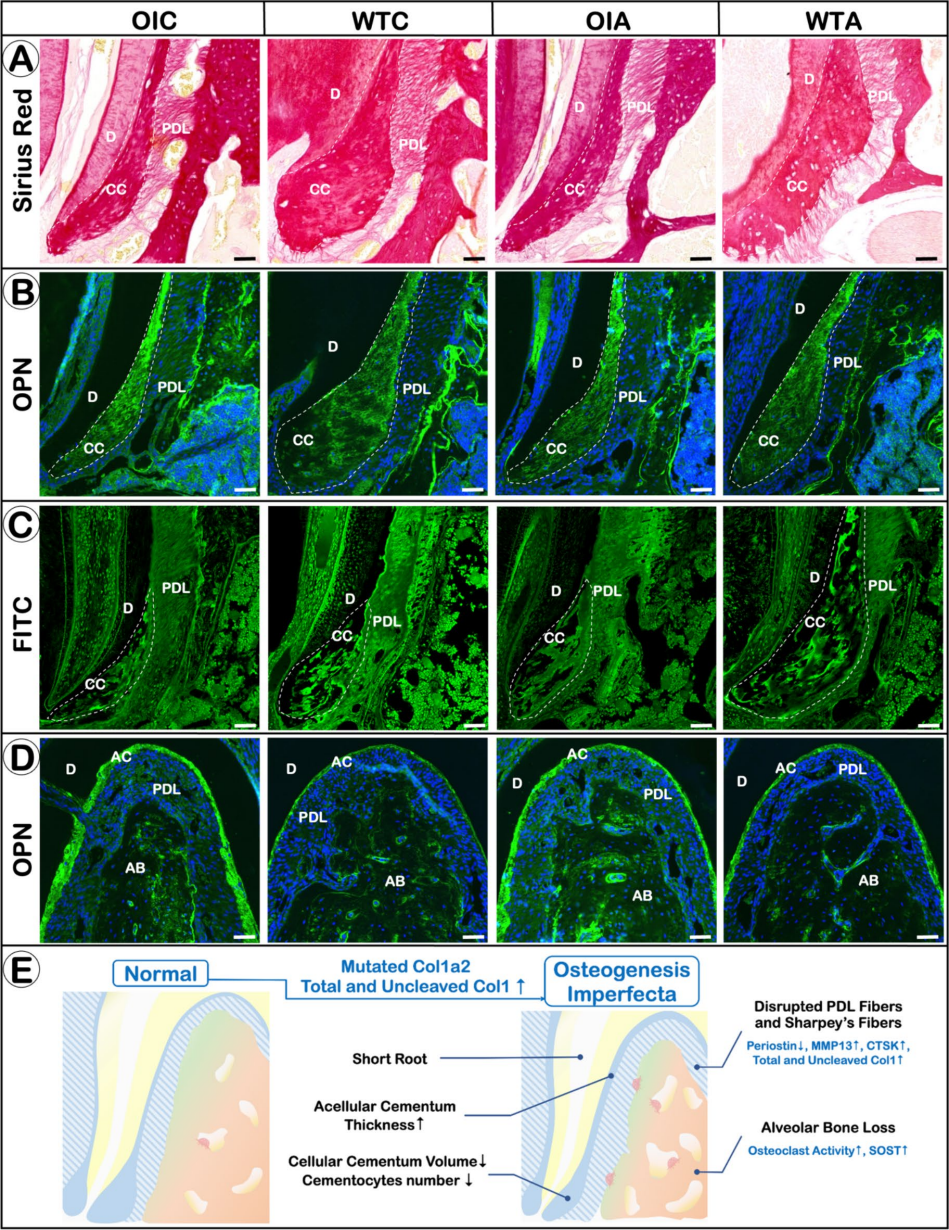
*Oim* ^−/−^ mice also exhibit defects in cementogenesis. Representative images of Sirius red staining (**A**), OPN immunostaining (**B**), FITC staining (**C**) and OPN immunostaining in the cementum (**D**). (**E**) Schematic diagram to show periodontal defects seen in the *oim*^*−/−*^ mice.

Immunofluorescence of osteopontin (OPN) revealed the cellular cementum in *oim* mice was smaller than in the WT, for both the 6-week and 3.5-month-old groups. In addition, FITC staining revealed a reduced cellular cementum and less cementocytes in the *oim* mice than in the WT animals, for both age groups. This defect in the cellular cementum in *oim* mice could be partially rescued by alendronate administration (Fig. 5A,B,C,E; Fig. S5 A,B,C).

Immunofluorescence of OPN also showed the presence of a thicker acellular cementum in *oim* animals, than in the WT for both age groups, and this could be partially rescued by alendronate administration (Fig. 5D; Fig. S5 D).

## Discussion

OI is a heritable disease of the connective tissue, which varies in severity depending on etiological mutations. Most patients with OI have mutations in genes for type I collagen alpha chains, COL1A1 and COL1A2^1–3^. Individuals with OI often require orthodontic treatment due to a higher prevalence of craniofacial and dental abnormalities, including midface deficiency, class III malocclusion, posterior and anterior crossbite, open bite, and missing teeth^6,7^.

The original classification by Sillence et al. (1979) divided OI into four types and is still the most widely used classification to date^36^. The classification has since been expanded to include at least seven types of OI^1,3^. Bone fragility is the most important characteristic of all types of OI, with its severity increasing in the following order: type I < types IV, V, VI, VII < type III < type II^3^. Type I – IV are all caused by mutations in *COL1A1* or *COL1A2*, while Type V – VII are associated with mutations in other genes^1,3^. For example, inactivating mutations in the gene for cartilage-associated protein (CRTAP) cause OI type VII in humans^1,32^. As our knowledge of the biological basis of this disease evolves, various transgenic mouse models have been developed for studying the pathogenesis and clinical phenotypes of the different types of OI^2,19,37–40^.

The periodontal ligament (PDL) is a group of specialized connective tissues which essentially anchors the cementum of a tooth to the alveolar bone. The PDL contains an abundant vascular network and a heterogeneous cell population including fibroblasts, osteoblasts, osteoclasts and mesenchymal stem cells. The PDL plays a critical role in responses to mechanical stress, and in maintaining the homeostasis of the periodontium^41,42^. Specifically, the PDL cells perceive mechanical signals caused by occlusion and mastication and respond by signaling to the progenitor cells of the periodontium, inducing differentiation into osteoblasts and osteoclasts^43^.

Entheses (insertion sites, osteotendinous junctions, osteoligamentous junctions) are sites of stress concentration at the region where tendons and ligaments attach to bone^44^. There are two types of entheses, fibrous entheses and fibrocartilaginous entheses. At fibrous entheses, the tendon or ligament attaches either directly to the bone or indirectly via the periosteum, and there is no evidence of cartilage differentiation as seen in fibrocartilaginous entheses^44^. The PDL is a type of fibrous entheses. Using SOST–GFP transgenic mice at the age of 3 weeks and 3 months, Lee et al. identified CD146 + cells adjacent to CD31 + vasculature at PDL-bone entheses; and expression of chondroitin sulfate proteoglycan 4 (CSPG4), osterix (OSX), and bone sialoprotein (BSP) at the PDL-bone and PDL-cementum entheses^45^. This indicates that biophysical cues resulting from development and function can regulate the recruitment and differentiation of progenitor cells in the PDL toward osteo- and cemento-blastic lineages at the PDL–bone and PDL–cementum entheses^45^.

PDL cells are thought to be the transducers of orthodontic loads^42,46,47^. OTM occurs when orthodontic forces are converted into biological events, where progenitor cells in the periodontium differentiate into osteoclasts and osteoblasts, thereby causing bone resorption and apposition, respectively^48,49^ Studies have shown that PDL-bone and PDL-cementum entheses at widened and narrowed PDL-spaces, following experimental tooth movement, express a high level of bone and neurovascular markers including OSX, BSP, protein gene product 9.5 (PGP9.5), and CD146, indicating active remodeling at these sites^50^. However, it remains largely unknown how the PDL entheses in OTM would be affected in the condition of OI.

To date, no studies have investigated the periodontal condition in patients with OI, however, a very recent study using a murine model of OI did identify significant periodontal defects associated with disease mutations^32^. In humans, inactivating mutations in the gene for cartilage-associated protein (CRTAP) causes OI type VII, with a phenotype that can include craniofacial defects. An in-depth examination of the *Crtap*^*−/−*^ murine model of OI revealed a series of periodontal dysfunction including decreased alveolar bone volume and mineral density, increased PDL space, ectopic calcification within the PDL, bone-tooth ankylosis, altered immunostaining of extracellular matrix proteins in bone and PDL, increased pSMAD5, and more numerous osteoclasts on alveolar bone surfaces. Although mouse lines have been developed to study other types of OI (e.g., *oim* model for Type III OI), detailed and quantitative analyses of periodontal tissues are lacking in those studies^51–54^. Considering that the collagen in *oim* mice exhibits a dramatic reduction of resistance against tensile stress^54–57^, defects of PDL entheses in *oim* mice are very likely in response to physiologic loads as well as orthodontic forces.

PDL-alveolar bone and PDL-cementum entheses exhibit active remodeling during physiological loading and orthodontic forces, but their roles under conditions seen in OI patients remains largely unknown. Bisphosphonates are commonly used for treating OI patients as they inhibit osteoclasts, decreasing bone resorption^1,3,12^. However, our knowledge in terms of how alendronate affects PDL entheses in OI is also far from complete. Therefore, the aim of this study is to determine the biological responses of the PDL entheses in a mouse model of OI under physiologic loads.

In addition to first observing a smaller body size, we also identified a smaller skull size and altered craniofacial skeletal phenotype in the *oim* mice. Quantitative micro-CT analysis confirmed a statistically significant reduction in several absolute interlandmark distances, including skull length, cranial height/width/length, nose length, nasal length, and frontal length. This is consistent with a previous study using *oim* mouse model^51^. Additionally, Xu et al. reported that cartilage-associated protein knockout (*Crtap*^*−/−*^) mice, a mouse model of OI type VII, also exhibited a brachycephalic skull shape with the fusion of the nasofrontal suture and facial bones, resulting in midface retrusion and Class III dental malocclusion^32^. *Oim* mice also showed several dental defects when compared to the WT group, including decreased alveolar bone volume, decreased cellular cementum volume, decreased dentin volume, and increased acellular cementum thickness. Loss of *Crtap* also led to similar dental defects^32^. In addition, the dental roots of *oim* mice were significantly shorter than those observed in WT mice.

Next, we specifically examined the periodontal tissues. There was a sharp reduction in the alveolar bone mass in *oim* mice, primarily resulting from increased osteoclastic bone resorption and increased SOST activity, associated with inhibition of bone formation^33–35^. Consequently, the bone formation rate in *oim* mice were much lower compared to the rate seen in the WT mice group. With alendronate treatment, the osteoclast number were greatly reduced in *oim* animals. However, treatment with alendronate did not show significant effects on SOST expression or mineral apposition rate.

In this study, *oim* mice showed severe disruption of the PDL fibers, and a loss of Sharpey’s fibers at the PDL-bone entheses. On one hand, the catabolism of PDL was increased, as revealed by elevated MMP13 and CTSK expression. On the other hand, the quality of the PDL fibers is defective due to a large accumulation of uncleaved Col I, indicating decreased activity of the cleavage enzyme. Similar periodontal defects and increased levels of uncleaved Col1 were also found in bone morphogenetic protein 1 (*Bmp1*) and related protease tolloid like 1 (*Tll1*) double knockout mice, resulting in a phenotype which mimics human type VIII OI^58^.

Our analysis showed that the cellular cementum volume was sharply decreased in *oim* mice, while the acellular cementum was thicker, relative to WT mice. This agrees with the cementum phenotype seen in the *Crtap*^*−/−*^ mouse model^32^. The mechanism inducing this change in the cementum of *oim* mice is still unclear.

## Conclusion

This study, for the first time, characterized periodontal defects in *oim* mice, detailed craniofacial defects, and demonstrated the effect of alendronate-treatment in partly restoring these defects. Since patients with OI have a higher prevalence of craniofacial and dental abnormalities and therefore require orthodontic treatment to achieve proper occlusal function, a better understanding of periodontal biology in patients with OI is important for clinicians to provide better prediction of prognosis and adjust treatment strategies.

## Electronic supplementary material

Below is the link to the electronic supplementary material.

Supplementary Material 1

## Data Availability

All data generated or analyzed during this study are included in this published article and its supplementary information files.
