# Supplementary material for: Alendronate partially rescues the periodontal defects in OIM mouse model of osteogenesis imperfecta
Source: Sci Rep. 2025 Jan 2;15:88. doi: 10.1038/s41598-024-84756-8 (PMC11695738; doi:10.1038/s41598-024-84756-8)
Supplement: Supplementary file 1 — Supplementary Material 1 [file 41598_2024_84756_MOESM1_ESM.docx]

**Supplemental data**

**Figure S1** Quantification of the enamel volume (A) and PDL volume (B) for the 6-week old and 3.5-month old groups of *oim* and WT animals. n = 4 - 5; Two-way ANOVA with Tukey’s post-hoc test, *P<0.05.

**Figure S2** *3.5-Month Old* *Oim^-/-^ Mice Display Alveolar Bone Defects which are Partially Improved by Alendronate Treatment.* (**A**) Representative Masson’s trichrome staining of mandibular first molars. (**B**) TRAP staining and quantification of the percentage of osteoclast positive surface versus bone surface (OcS/BS). n = 4 - 5; Two-way ANOVA with Tukey’s post-hoc test, *P<0.05, **P<0.01. (**C**) SOST immunostaining. (**D**) Representative images showing fluorescence labeling; Calcein (green label) and alizarin complexone (red label) were injected 7 days and 2 days before sacrifice, respectively.

**Figure S3** *3.5-Month Old* *Oim^-/-^ Mice Display a Severe PDL Phenotype which can be Partially Improved by Alendronate Treatment.* Representative images of Sirius red staining (**A**), immunofluorescent staining of periostin (**B**) and FITC staining (**C**).

**Figure S4** *Molecular Changes in The Periodontium of Oim^-/-^ Mice at 3.5-Months of Age.* Representative images of immunofluorescent staining of total Col1 (**A**), uncleaved Col1 (**B**), MMP13 (**C**) and CTSK (**D**) in the PDL and alveolar bone tissue.

**Figure S5** *Oim^-/-^ Mice Also Exhibit Defects in Cementogenesis at 3.5-Months of Age.* Representative images of Sirius red staining (**A**), OPN immunostaining (**B**), FITC staining (**C**) and OPN immunostaining in the cementum (**D**).

**Figure S1**

**
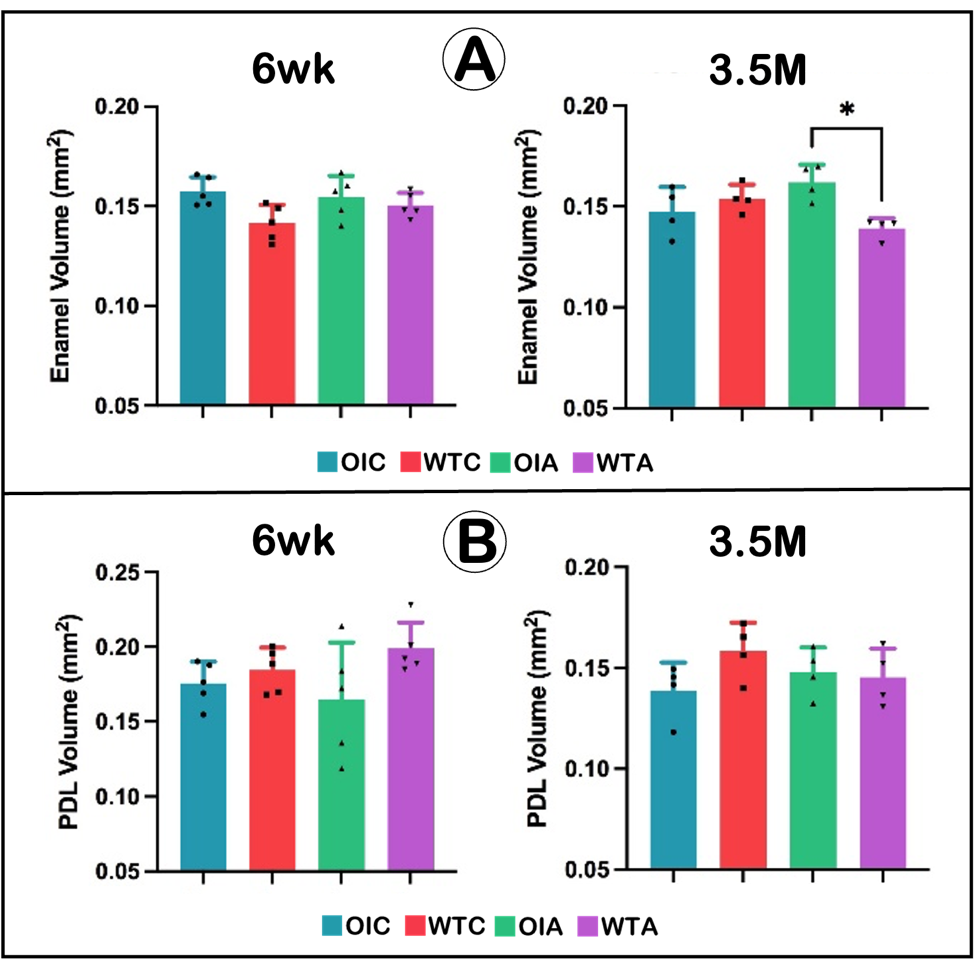
**

**Figure S2**

**
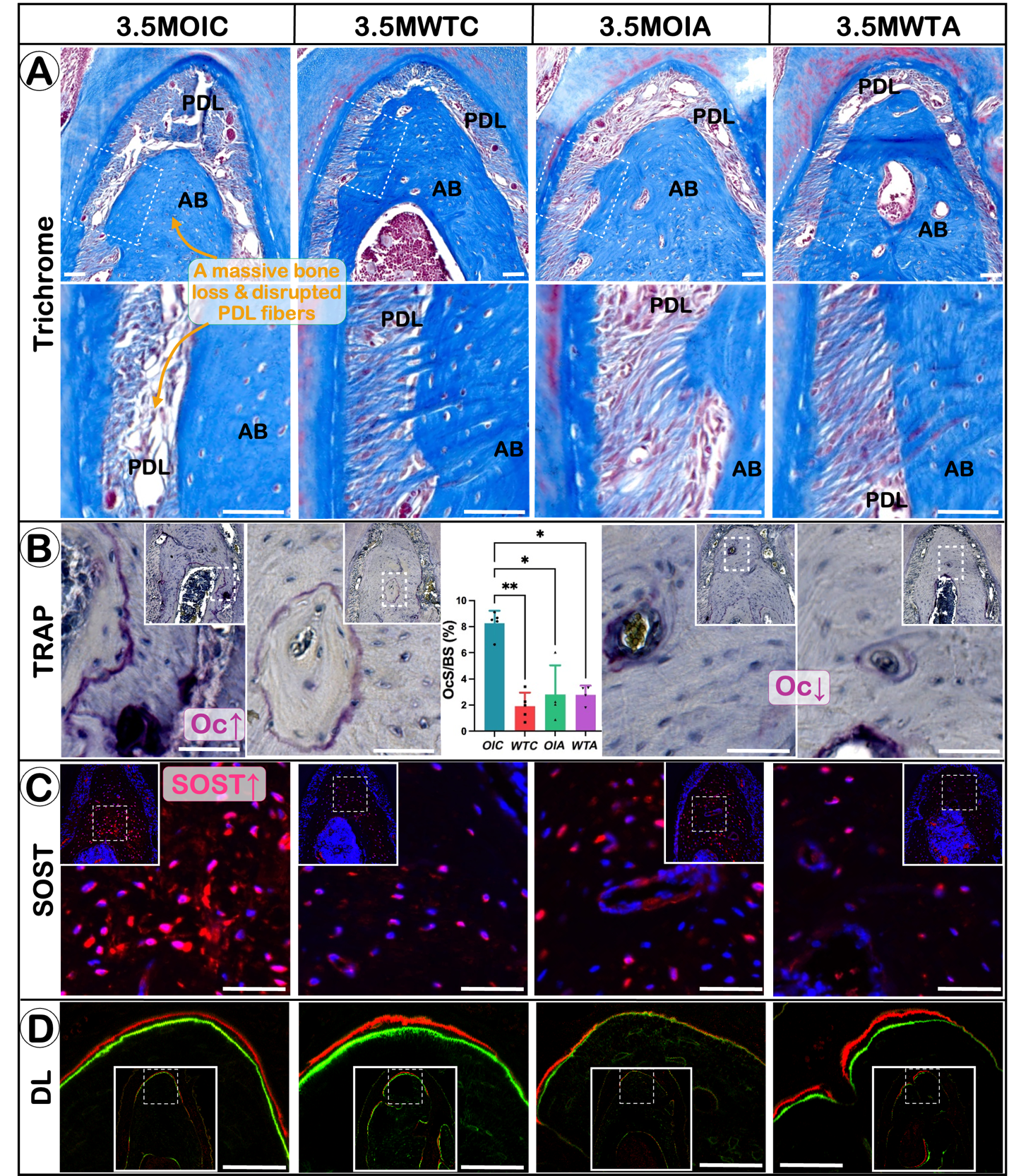
**

**Figure S3**

**
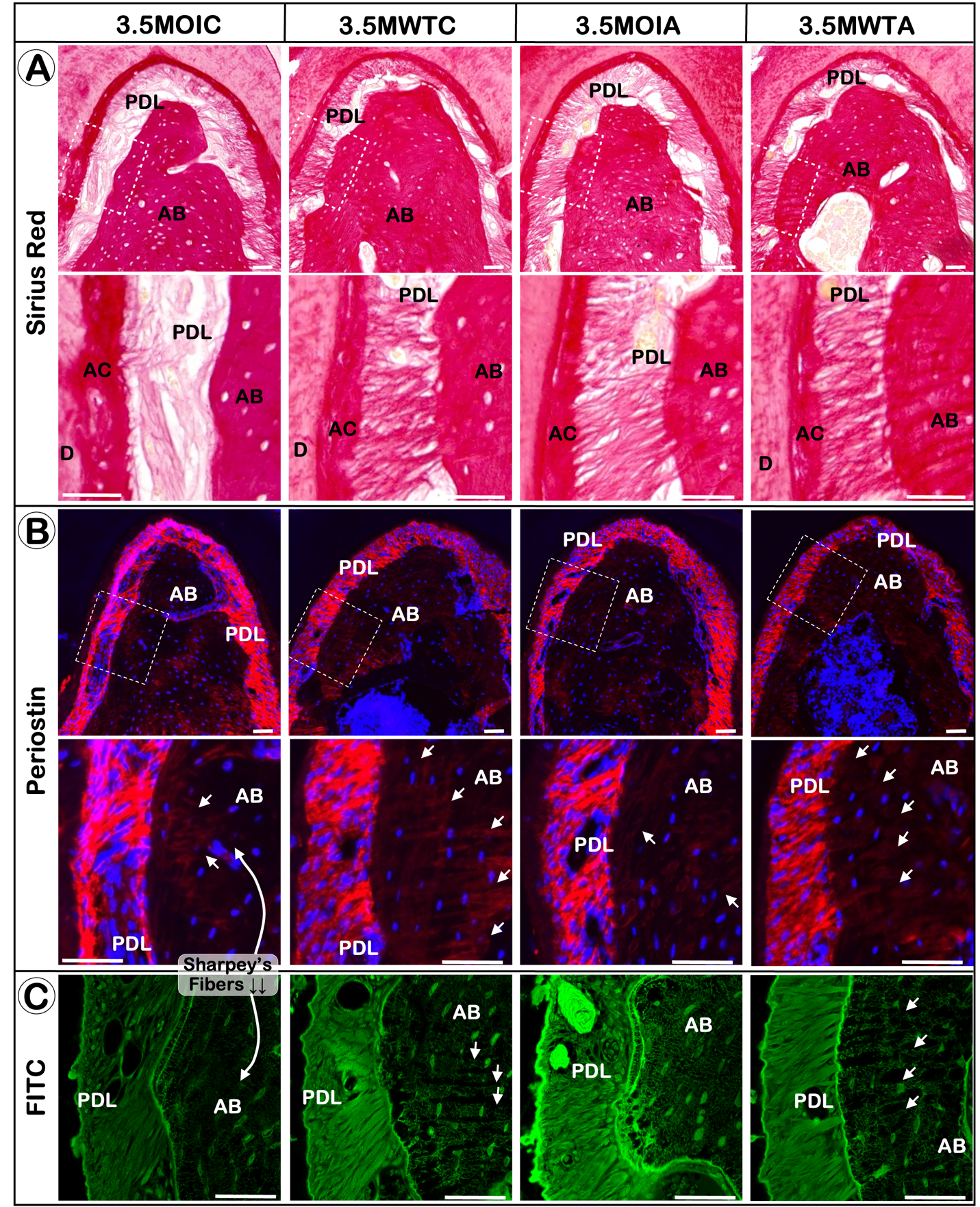
**

**Figure S4**

**
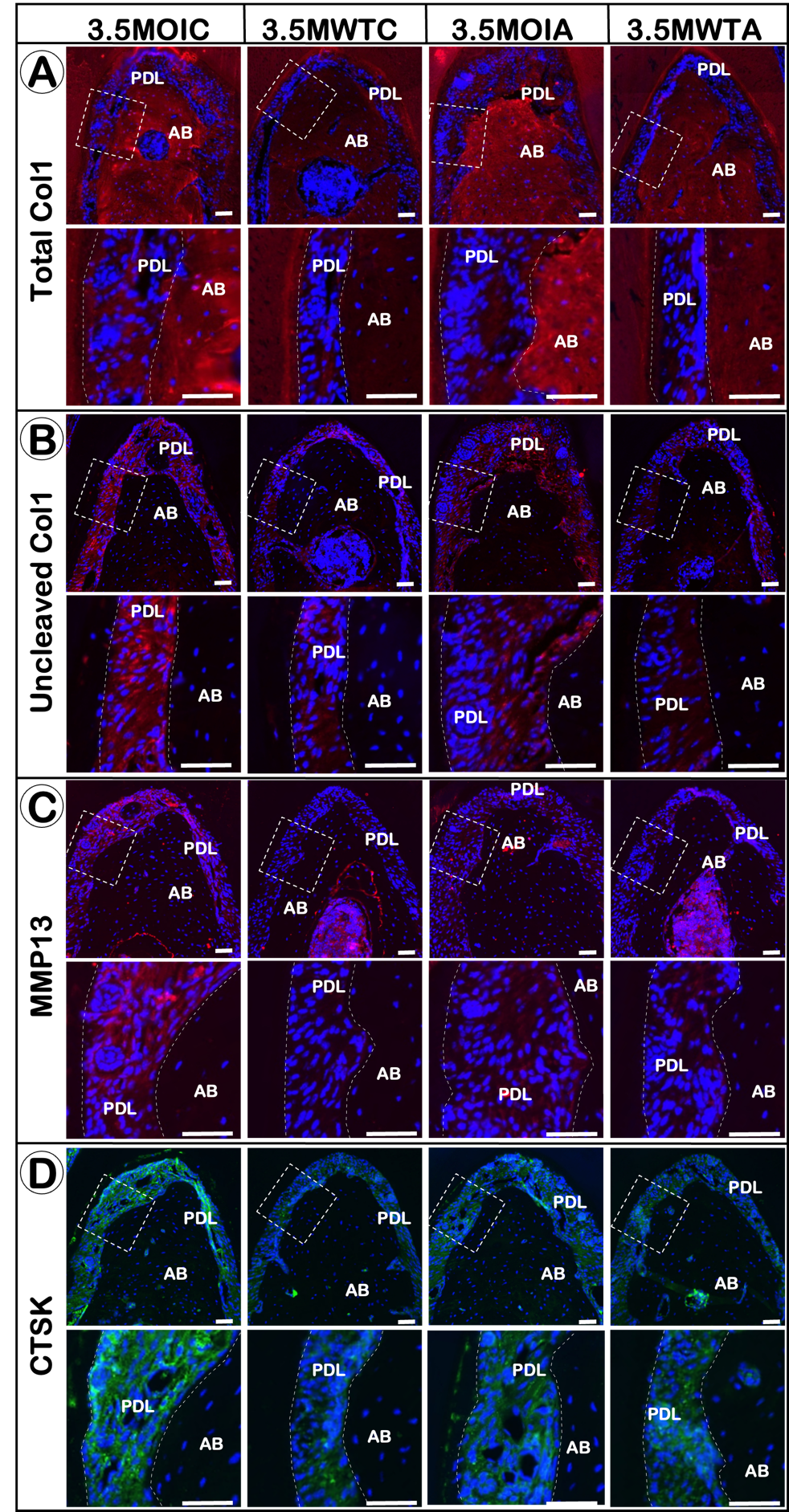
**

**Figure S5**

**
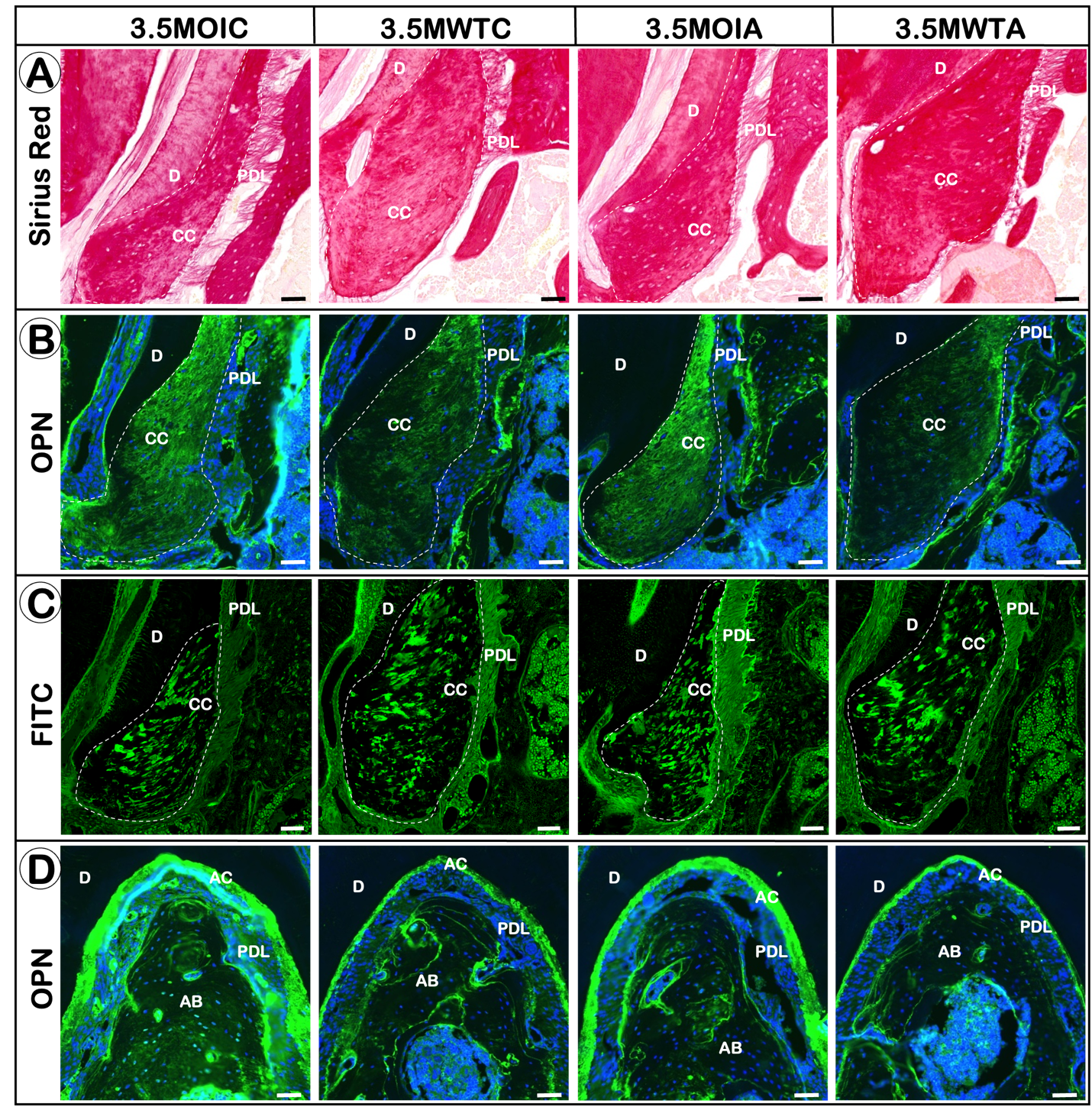
**
